# Thyroid cancer diagnosis by Raman spectroscopy

**DOI:** 10.1038/s41598-020-70165-0

**Published:** 2020-08-07

**Authors:** Marco Sbroscia, Michael Di Gioacchino, Paolo Ascenzi, Pierfilippo Crucitti, Alessandra di Masi, Isabella Giovannoni, Filippo Longo, Davide Mariotti, Anda Mihaela Naciu, Andrea Palermo, Chiara Taffon, Martina Verri, Armida Sodo, Anna Crescenzi, Maria Antonietta Ricci

**Affiliations:** 1 Dipartimento di Scienze, Universitá degli Studi Roma Tre, Rome, Italy; 2 Unit of Thoracic Surgery, Campus Bio-Medico University, Rome, Italy; 3 Pathology Unit, Campus Biomedico University Hospital, Rome, Italy; 4 Unit of Endocrinology and Diabetes, Campus Bio-Medico University, Rome, Italy; 5 Present Address: Dipartimento di Fisica, Sapienza Universitá di Roma, Rome, Italy

**Keywords:** Biophysics, Cancer, Endocrinology

## Abstract

Over the last 50 years, the incidence of human thyroid cancer disease has seen a significative increment. This comes along with an even higher increment of surgery, since, according to the international guidelines, patients are sometimes addressed to surgery also when the fine needle aspiration gives undetermined cytological diagnosis. As a matter of fact, only 30% of the thyroid glands removed for diagnostic purpose have a post surgical histological report of malignancy: this implies that about 70% of the patients have suffered an unnecessary thyroid removal. Here we show that Raman spectroscopy investigation of thyroid tissues provides reliable cancer diagnosis. Healthy tissues are consistently distinguished from cancerous ones with an accuracy of $\sim$ 90%, and the three cancer typology with highest incidence are clearly identified. More importantly, Raman investigation has evidenced alterations suggesting an early stage of transition of adenoma tissues into cancerous ones. These results suggest that Raman spectroscopy may overcome the limits of current diagnostic tools.

## Introduction

Detection of nodular thyroid pathology is increasing worldwide paired with an increased number of thyroid cancer diagnosis^1^. Whether this “epidemic” spread is real or is due to an exaggerated use of imaging tools in clinical practice is object of a vivid debate within the scientific community. Anyway, management of patients with thyroid nodule causes a clinical problem and a social burden as well^2^. To avoid overdiagnosis and overtreatment of these patients, physicians need additional information about the biology of each thyroid nodule. In particular there is need for a tool able to distinguish between benign and malignant follicular lesions and between indolent and aggressive tumors^3^. The genomic profile of thyroid tumors has been extensively investigated^4^ and several immunocytochemical and molecular markers have been proposed for a more accurate classification of thyroid nodules^5^. However, none of these markers reaches a reliable sensibility and specificity to be routinely used in clinical setting^6, 7^. Moreover, although the major genetic alterations of thyroid tumors are known, only little information, if any, is available about changes in protein, lipids and other cellular constituents due to thyroid oncogenesis.

To correctly manage patients with thyroid nodules and to avoid unnecessary surgery, we strongly need a method of analysis that overcomes the limits of current diagnostic tools, by identifying specific tumoral markers, possibly with high spatial resolution and low impact on the patient. Cells and tissues are characterized by a specific biochemical composition. Each pathology or cellular abnormality is accompanied by biochemical molecular changes. Optical and spectroscopic techniques that correlate the biochemical composition, molecular structure, and their variations with the morphological alterations represent a powerful diagnostic and potentially clinical tool^8^.

Among the experimental techniques available for chemical-physical studies of matter at the molecular level, Raman spectroscopy (RS) is a good candidate for our needs. This technique offers a wealth of molecular information, while being not invasive, and achieving spatial resolutions as high as a few μm in micro-Raman apparatuses. RS probes the vibrational dynamics of the sample, through a fast and not destructive analysis. It does not require any sample preparation and can consequently be applied in vivo. For these characteristics it has been recently applied on a variety of biological and medical samples to identify specific metabolic states of cells, tissues and bacteria, and in particular to differentiate between healthy and cancer tissues^9–12^. Preliminary studies on thyroid tissues by RS suggest that this technique may have high impact in thyroid cancer diagnosis^13–18^.

**Figure 1:**
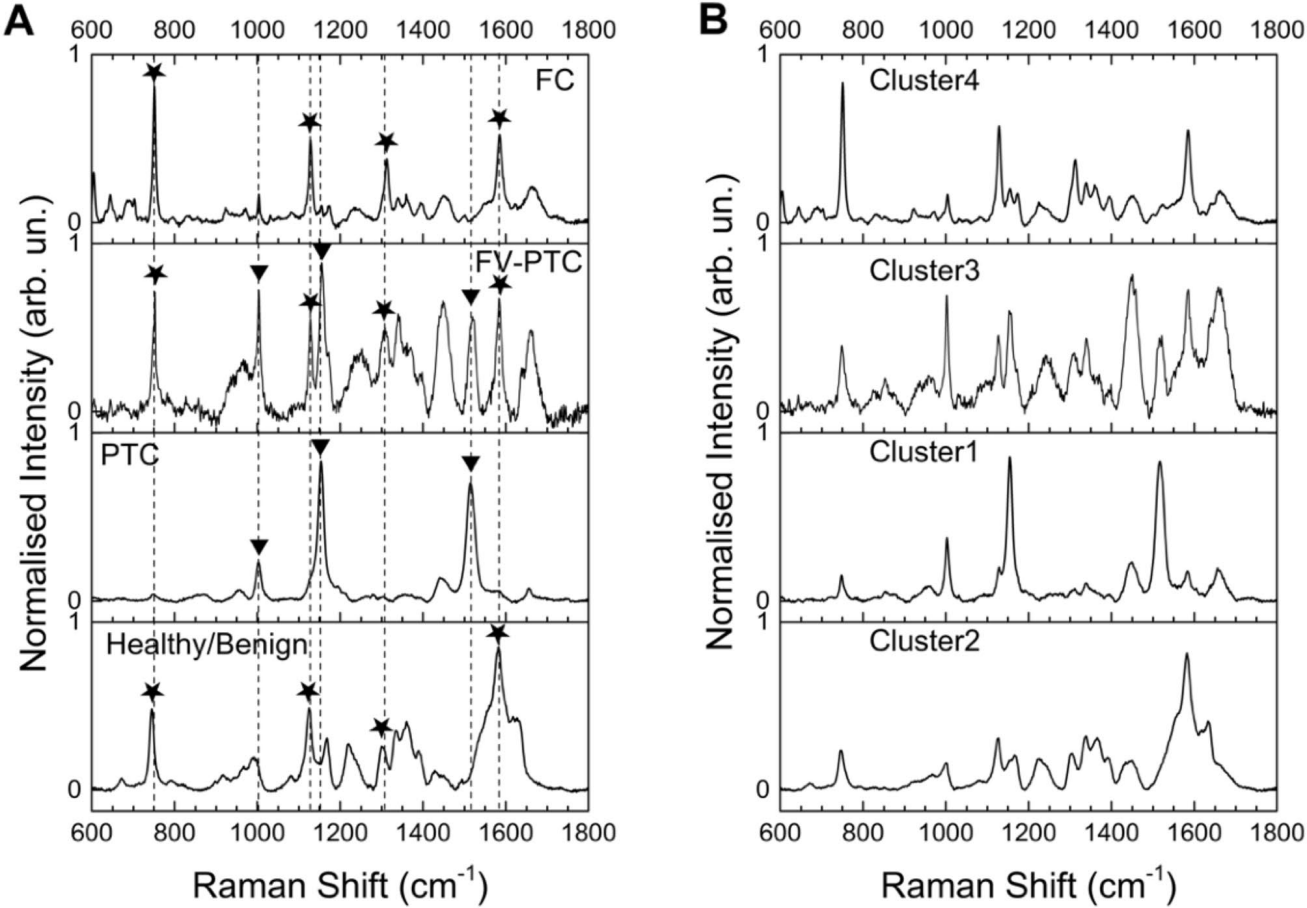
Raman spectra. (**A**) Typical Raman spectra of the examined thyroid tissues, labelled according to the histology report. Stars label the Raman characteristic peaks of cytochrome *c*, triangles those of carotenoids. The black vertical dashed lines allow an easier comparison among the spectra. (**B**) Centroids Raman spectra for the four identified clusters by K-means analysis.

In a RS experiment a laser beam is focalized on the sample and the scattered light is analyzed in frequency. Typical spectra, after background subtraction, are shown in Fig. 1A. These are characterized by peaks and bands with frequency centered at the vibrational frequency of the intramolecular bonds characteristic of the main sample components.

We have applied RS augmented by cluster analysis to investigate histological samples from human thyroid. Assignement of the RS peaks to carotenoids or cytochrome *c* in different amounts has allowed to distinguish healthy tissues from cancerous ones and to discriminate for the first time among the three categories of carcinoma with larger incidence, namely Papillary carcinoma (PTC), follicular variant of Papillary carcinoma (FV-PTC) and Follicular carcinoma (FC). We have also analyzed samples labelled after histology as follicular adenoma (FA). We found that these samples are spread over four categories (reported above) by the cluster analysis. Subsequent more specific analyses of protein expression have confirmed an anomalous level of tumoral or benign molecular markers in the adenoma samples labeled in the non healthy/benign categories by RS. All the samples analyzed by RS are reported in a table as Supplementary information.

## Results

Figure 1A shows, from bottom to top, the Raman fingerprint region ($600{-}1{,}800 \, {\hbox {cm}}^{-1}$) of four samples with a clear histology diagnosis, namely Healthy/Benign (average of the spectra of samples Hea49 and Hea64), PTC (average of the spectra of samples TIR51 and TIR58), FV-PTC (sample TIR56) and FC (average of the spectra of samples TIR47 and TIR55) carcinoma tissues. Each spectrum has been normalized to its integrated intensity. We notice that each tissue has its own characteristic spectral lineshape. According to Rau et al.^15^ and references therein, this spectral range shows the bands ascribed to proteins, lipids and aminoacids. Here we will not report a detailed assignment of each band to a particular biomolecule, as this is sometimes tricky and not relevant to our aim. We will instead put the attention on the distinctive set of peaks which characterize each histological sample. We notice that the spectrum of the healthy tissues is dominated by an intense, broad and structured band centered around $\sim 1,600 \, {\hbox {cm}}^{-1}$, possibly due to the presence in the sample of amide I and lipids^19, 20^. The sharpest structure of this band falls at $1,582 \, {\hbox {cm}}^{-1}$ and along with the bands at 747; $1{,}120 \div 1{,}128$, and $1{,}301 \, {\hbox {cm}}^{-1}$ represets the fingerprint of cytochrome *c*^20, 21^. This is a protein localized in the mitochondrial intermembrane space under normal physiological conditions. The spectrum of a classical papillary carcinoma (labeled PTC in Fig. 1A) is totally different and unambigously shows the presence of carotenoids (bands at 1,003, 1,155 and $1{,}516 \, {\hbox {cm}}^{-1}$), not present in healthy tissues, as already reported in the literature^10, 14, 15, 22, 23^. In the follicular carcinoma spectrum (labeled FC in Fig. 1A) we observe a marked enhancement of the cytochrome *c* bands. As a matter of fact, the broad band at $\sim 1,600 \, {\hbox {cm}}^{-1}$ that dominates the Healthy/Benign spectra is barely visible in this case, after spectral intensity normalization. Interestingly, the spectrum of the Papillary Follicular carcinoma (labeled FV-PTC in Fig. 1A) looks like a superposition of the PTC and FC spectra, showing the fingerprints of carotenoids along with a clear enhancement of the bands ascribed to cytochrome *c* with respect to the spectra of Healthy/Benign tissues.

The presence of carotenoids in both PTC and FV-PTC carcinoma is peculiar and worthy of interest. The cellular process of carotenoids uptake (not directly synthetized by human organisms) and their proper identifications is under biochemical investigation.

**Figure 2:**
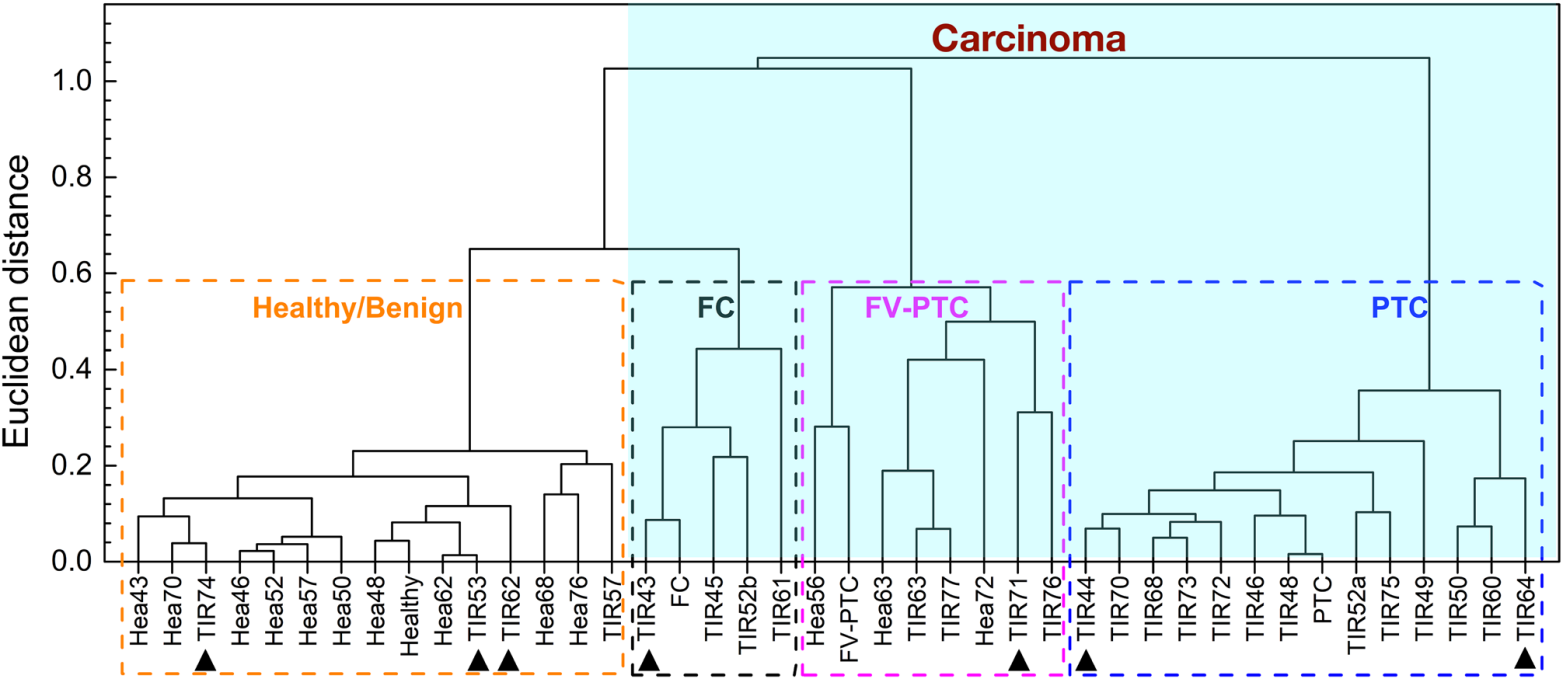
Agglomerative hierarchical clustering analysis. Dendrogram of the Raman spectra of human thyroid tissues, as extracted from the AHCA analysis. Individual samples are represented by the label Hea (for healthy), or TIR (for not healthy) followed by the patient anonymous ID code. Those plotted in Fig. 1A are labelled healthy, FC, FV-PTC and PTC, respectively. Dashed squares identify the four clusters, namely healthy/benign (orange), Follicular carcinoma or FC (black), follicular variant of papillary carcinoma or FV-PTC (magenta) and papillary carcinoma or PTC (blue). The arrows indicate the adenoma samples. All samples within the light blue shaded area are carcinomas.

As Raman spectroscopy is able to detect the differences between healthy and cancerous tissues, it may be considered as a promising tool for in situ cancer diagnosis. We have analyzed a total of 46 histological samples of human thyroid tissues: 8 samples diagnosed as adenomas, including one inadequate (TIR59) for RS and not further considered for statistical analysis; 15 healthy ones and 23 carcinomas ones, namely 15 PTC, 4 FV-PTC, 3 FC and 1 Hyalininzing Trabecular Tumor (HTT). All samples are labelled as TIR or Hea, followed by the patient anonymous ID code.

Within each histology class, spectra may differ for noise, background or fluorescence contributions, although keeping unaltered the characteristic fingerprints commented above. This observation suggests that the experimental spectra can be analyzed on quantitative grounds by cluster analysis, after backgrounds and fluorescence subtraction. This aims at classifying each Raman spectrum into homogeneous groups, based on its characteristic features, in other words on the presence and relative intensity of specific bands. The analysis has been performed following two distinct approaches (see “Methods”).

The first approach, called agglomerative hierarchical clustering analysis (AHCA), builds a dendrogram of the entire data set, by iteratively selecting pairs of the closest spectra or groups of spectra as a function of their Euclidean distance. The results of this analysis are reported in Fig. 2, where each sample is represented by means of the label Hea or TIR (depending on whether the tissue has received a healthy or not healthy histological diagnosis) followed by a progressive number. This figure tells us that our spectra are consistent with a classification of the dendrogram into 4 clusters, assuming a distance threshold of 0.58 compared to a maximum distance of 1.05. The four clusters have been labelled according to the diagnosis of the four spectra shown in Fig. 1A, a priori selected as the most representative of each histology report, namely the less noisy with a clear and confirmed diagnosis. Healthy/Benign and PTC tissues are very well distinct each other and have the smallest dispersion in terms of Euclidean distance within each cluster. Indeed, the smaller is the dispersion of the Euclideian distances, the better is the homogeneity of the group: in the present case Healthy/Benign and PTC tissues are the most homogeneous. The other two clusters (FC and FV-PTC) are more dispersed, although equally distinguishable from the others. The FC cluster is the closest to the Healthy/Benign one, as both are characterized by the presence of cytochrome *c*, and distinguished only on the basis of its Raman fingerprint relative intensity. The FV-PTC tissues fall between FC and PTC clusters, as predictable from copresence of carotenoids and citochrome *c* peaks in their Raman spectra.

**Figure 3:**
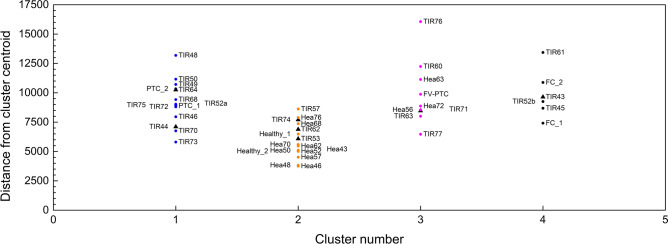
K-means analysis. Samples distribution within the four KM clusters, as a function of their Euclidean distance from the associated centroid. The same labels and colours as in Fig. 2 have been used. Black triangles refer to adenomas.

The second unsupervised statistical approach considered is called K-means (KM): its results are reported in Fig. 3. This confirms that four clusters properly describe our data set. Clusters 1, 2, 3, 4 contain samples from the histology reports of PTC , Healthy/Benign, FV-PTC and FC, respectively. The agreement between the clustering results of the two approaches is very good, as we notice only one difference, concerning sample TIR60, which switches from a Raman diagnosis of PTC to that of FV-PTC. Fig. 3 shows that healthy/benign tissues spectra have on the average the shortest distance from their centroid and the smallest dipersion. On the contrary the spectra of FV-PTC tissues are the most dispersed, possibly due to a different balance of concentration between carotenoids and cytochrome *c* in each sample. Table 1 reports the distance matrix among the clusters centroids. Cluster 1, showing the largest centroid distance from the others, is readily isolated (its Raman spectrum is dominated by the carotenoid fingerprint), while discrimination among the others requires consideration of more spectral details.

**Table 1:** Euclidean distance between the cluster centers.

	Cluster 1	Cluster 2	Cluster 3	Cluster 4
Cluster 1	0	24,611	19,319	24,211
Cluster 2	24,611	0	13,539	14,249
Cluster 3	19,319	13,539	0	14,117
Cluster 4	24,211	14,249	14,117	0

**Table 2:** Confusion matrix, based on 38 samples (healthy and carcinomas tissues). This shows that RS has identified 1 False negative diagnosis among the 15 negative Medical diagnosis and 3 different samples among the 23 with a positive diagnosis, which fall in a cluster different from those expected on the basis of the medical diagnosis.

Total of 38 samples	Raman	Classification	Accuracy
Medical diagnosis	True negative 12	False positive 3	
	False negative 1	True positive 22	0.90

KM analysis returns also the Raman spectrum calculated for each cluster centroid: these are reported in Fig. 1B, for direct comparison with the prototypical spectra of the four classes of tissues. The similarity of the spectra reported in Fig. 1A,B is astonishing and proves that our clustering is solid.

Based on these results, we have computed a confusion matrix, providing the accuracy of our analysis in discriminating between healthy/benign and pathological tissues, regardless of the specific pathology. As reported in Table 2, our accuracy is of $\sim\, 90\%$.

**Table 3:** Confusion matrix, based on 45 samples (including adenomas).This shows that RS has identified 1 false negative diagnosis among the 15 negative medical diagnosis and 7 different samples among the 29 with a positive diagnosis, which fall in a cluster different from those expected on the basis of the medical diagnosis.

Total of 45 samples	Raman	Classification	Accuracy
Medical diagnosis	True negative 15	False positive 7	
	False negative 1	True positive 22	0.82

Finally, we have applied the same analysis including the spectra of the samples classified as adenoma by the histological diagnosis. Their position is highlighted by arrows in the AHCA dendrogram of Fig. 2 and in the distributions of Fig. 3. These figures show that the spectra of adenoma tissues spread over all four clusters defined by healthy and cancerous tissues. This is a quite unexpected result, as adenomas are classified as benign tissues and therefore should fall into the healthy/benign cluster. Accordingly, the confusion matrix after inclusion of adenoma samples, reported in Table 3, returns an accuracy of the statistical analysis of 82%.

**Figure 4:**
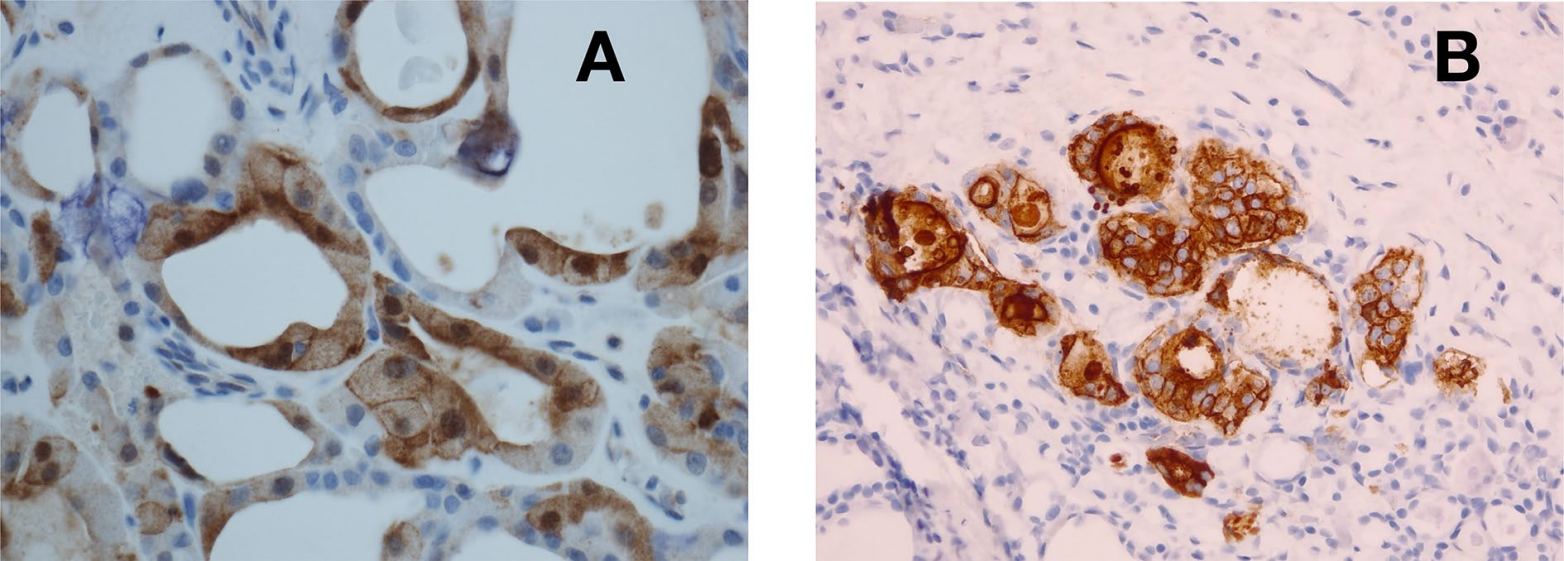
Immunohistochemistry images. Microscopic pictures of (**A**) TIR43 sample stained with immunohistochemistry for Galectin3 (high power field. Hematoxylin counterstained) and (**B**) TIR64 sample stained with immunohistochemistry for HBME1 (medium power field. Hematoxylin counterstained). Panel (**A**) shows a mosaic pattern with negative cells intermingled with cells showing positive reaction in cytoplasm as well as in nuclear matrix. Panel (**B**) shows strong positive reaction due to a microfocus of papillary carcinoma, within the follicular adenoma.

**Table 4:** Confusion matrix, based on 45 samples (including adenomas), after immunohistochemistry revision.This shows that RS has identified 1 false negative diagnosis among the 15 negative medical diagnosis and that the number of false positive diagnosis goes down to 3.

Total of 45 samples	Raman	Classification	Accuracy
Medical diagnosis	True negative 15	False positive 3	
	False negative 1	True positive 26	0.91

**Figure 5:**
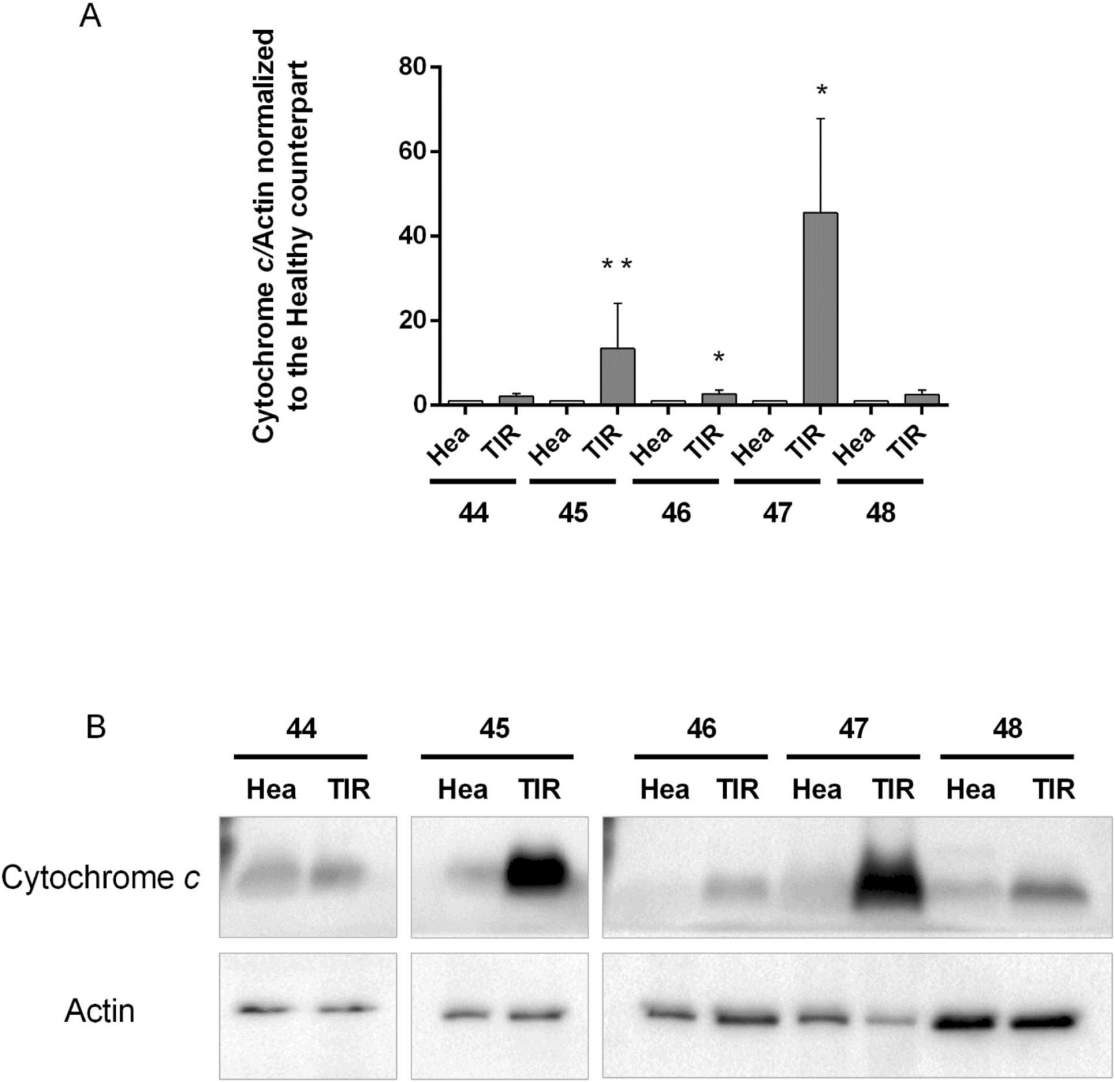
Biochemical analysis. Expression levels of cytochrome *c* have been assessed in thyroids of five patients; for each patient, the healthy (Hea) and pathological (TIR) slices have been analysed. (**A**) Levels of cytochrome *c* have been normalized to actin. Data represent the mean values ± SDs derived from three replicates normalized to healthy counterparts (Student’s *t* test, *P < 0.05, **P < 0.01 compared with control). (**B**) Exemplificative images of filters blotted with cytochrome *c* and actin primary antibodies. Images have been gathered at the same time.

After the RS analysis, four adenoma samples (TIR43, TIR44, TIR64, TIR71) falling within the cancerous clusters have been in depth reanalyzed as far as their expression of benignity and cancerous markers are concerned, by immunohistochemistry test. The lowering of benignity markers (CD56) or sometimes the onset of tumoral markers (Galectin3, HBME1) are revealed in all these cases. The immunohistochemistry images of TIR43 and TIR64 samples are shown in Fig. 4, as representative of the all four cases. These results could be symptomatic of early stages of progression of adenomas into a specific type of carcinoma. Such type of transformation has never been demonstrated and our Raman observation opens a new light about the malignant potential of thyroid FA and about the possibility to assess which of these lesions may constitute a clinical concern. Interestingly the confusion matrix, after this revision, gives an accuracy of $\sim$ 91%.

A few anomalies, visible in Figs. 2 and 3, deserve a comment. Sample Hea56 and sample Hea63 apparently fall into a wrong cluster, namely the FV-PTC cluster, but interestingly very close to their TIR counterpart (FV-PTC and TIR63,respectively). This may happen when the Raman spectrum of the healthy portion is collected from a tissue region too close to the pathologic one, where normal and altered cells are almost intermingled, giving the superposition of healthy and cancerous Raman spectra. In particular, the case of sample Hea72, falling within the FV-PTC cluster, may be due to the diffusion of carotenoids in normal parenchyma at the border of the PTC nodule, as already observed in previous Raman chemigram map of thyroid carcinomas^15^. This gives in the Raman spectrum the superposition of the fingerprint of carotenoids with fingerprint of healthy tissues (containing cytochrome *c*), thus explaining the “wrong” assignment obtained by Raman analysis.

Additionally, levels of cytochrome *c* have been further evaluated by biochemical analysis (Fig. 5) on a few cases. Indeed, only for patients 44, 46 and 48 (PTC), and 45 and 47 (FC) a sufficient amount of thyroid tissue was available to perform immunoblot analyses. As shown in Fig. 5, cytochrome *c* levels significantly increase in TIR45 (13.4-fold induction compared to its healthy counterpart Hea45; P < 0.01), TIR46 (2.7-fold induction compared to Hea46; P < 0.05), and TIR47 (45.6-fold induction compared to Hea47; P < 0.05). In agreement with Raman results, cytochrome *c* levels are similar in TIR and Hea slices of patients 44 and 48.

In conclusion, this study demonstrates the capability of Raman spectroscopy as diagnostic tool for thyroid cancer. Indeed through a multivariate statistical analysis of the spectra it is possible to readily separate healthy from cancerous tissues and in the majority of the cases to discriminate among the most common cancer typologies (see Supplementary information for details on the individual cases). Last but not least, our findings highlight the possible usefulness of Raman spectroscopy as a tool for in situ and early diagnosis of thyroid pathology.

## Methods

### Study enrollment

From January 2018 to January 2020, we enrolled 30 consecutive subjects (21 females and 9 males) with thyroid nodular pathology referred to the Endocrinology Unit of Campus Bio-Medico University Hospital. Their age distribution is centered at 45 years with a standard deviation of 13 years. All the study population had received a cytological diagnosis of indeterminate, suspicious or malignant lesion with a formal indication to surgery (total thyroidectomy) according to the international guidelines^24^. After signing the informed consent, these patients underwent total thyroidectomy at the Surgical Unit of the same Institution.

### Neck ultrasound and clinical evaluation

All subjects were submitted to thyroid US evaluation, using a frequency range of 10–12 MHz on a MyLab 50 (Esaote, Genova, Italy). US scan of thyroid gland and neck area were performed by 2 experienced endocrinologists at the Endocrinology Unit. Nodules were classified according to ACR TI-RADS risk stratification criteria^25^ without prior knowledge of the cytological results. When there was a disagreement, the endocrinologists conducted a separate session to reach an unified consensus. We collected demographic data, including age, gender, family history of thyroid cancer and, using the medical records, we evaluated the thyroid function and the presence of autoimmune thyroiditis.

### Thyroid tissues preparation

At the time of surgery, the removed specimens were immediately submitted unfixed to the Pathology Unit in an appropriately labelled container. The pathologist valued the gross anatomy of the samples and a tissue slice of about 1 cm wide $\times$ 1 cm length $\times$ 3 mm of thickness, was cut, including both healthy and neoplastic areas, avoiding surgical margins. The slice was snap frozen on a metallic cold-plate inside a cryostat. A 5 μm section was stained with haematoxylin/eosin in order to confirm the presence of healthy and neoplastic tissues. After this step, four consecutive cryostatic sections were cut at 30 μm of thickness, collected on separate slides and stored at ${-}\,20^\circ  \hbox {C}$ until the Raman evaluation. Our Raman study was exclusively performed on these frozen unfixed samples. The residual slices were defrosted, formalin fixed, and paraffin embedded with the paired surgical samples for definitive histology. Final diagnosis was reported in agreement with current edition of WHO classification of endocrine tumours^26^. Immunohistochemical analysis for Galectin3 (Gene Tex), CD56 (Agilent), and HBME1 (Agilent) was performed in each case on paraffin sections from neoplastic areas using an automatized immunostainer (Omnis, Agilent).

Nodules with diameter wider than 2 cm were submitted for biochemical analysis. In such cases, at the time of gross dissection, further slices of about $0.5 \times 0.5$ cm were cut from the surgical specimen, sampling both healthy and neoplastic tissue. Slices were snap frozen and stored at ${-}\,80^\circ \hbox {C}$ until biochemical evaluation.

### Raman spectra collection

Raman spectra have been collected by means of a Renishaw InVia Micro-Raman spectrometer. In this set-up unpolarised spectra are collected through a Leica DM2700 M confocal microscope equipped with a Leica 50$\times$ LWD and an Olympus 100$\times$ objective. The required high-contrast rejection for the elastically scattered light is provided by an holographic edge filter. A diffraction grating with 1,800 grooves/mm disperses the Raman inelastic scattering contribution providing a spectral resolution of about $1 \, {\hbox {cm}}^{-1}$. A Peltier cooled 1,024 $\times$ 256 pixel CCD detector collects the dispersed light. A solid-state diode laser source at 532 nm with a nominal output power of nearly 60 mW, has been used as excitation source. To prevent photo-damage, neutral density filters have been used for lowering the laser power at the sample. The spot size has been shrink down to few microns when necessary to isolate the contribution of cells (about ten microns in size) from the surrounding cytoplasm, thus reducing spectral interference. Spectra have been collected in the extended scan mode, covering the 100–$3{,}800 \, {\hbox {cm}}^{-1}$ frequency shift range. and accumulating 5 scans with an equivalent integration time of nearly 10 s per scan. Measurements have been collected on 5 points per sample. Wire (Renishaw) software has been used to collect the raw spectra. Wire and LabSpec software has been used to perform the preliminary data reduction (e.g. background and fluorescence subtraction), while Origin (OriginLab Corporation) software has been employed to perform the statistical analysis. After background and fluorescence subtraction, all the spectra have been normalised to their integral, in order to avoid any bias due to fluctuations of the experimental conditions.

### Cluster analysis of Raman spectra

In order to gain better confidence on the clustering analysis, we have selected two unsupervised multivariate statistical approaches to analyze the Raman spectra: the agglomerative hierarchical clustering analysis (AHCA) and the K-means one (KM). Both methods are based on the Euclidean distance among spectra, as a measure of their similarity/dissimilarity. In detail, in these analyses, spectra are represented by their distance with respect to an origin, thus generating a dissimilarity matrix. Among the possible choices for the spectral distance, we have chosen the Euclidean one, since it maximizes the similarities among the spectra. The choice to use these two methods was made to exploit their complementarity of hierarchical and non-hierarchical approaches and consequently to give solidity to the obtained results^13, 14, 27–29^.

**Figure 6:**
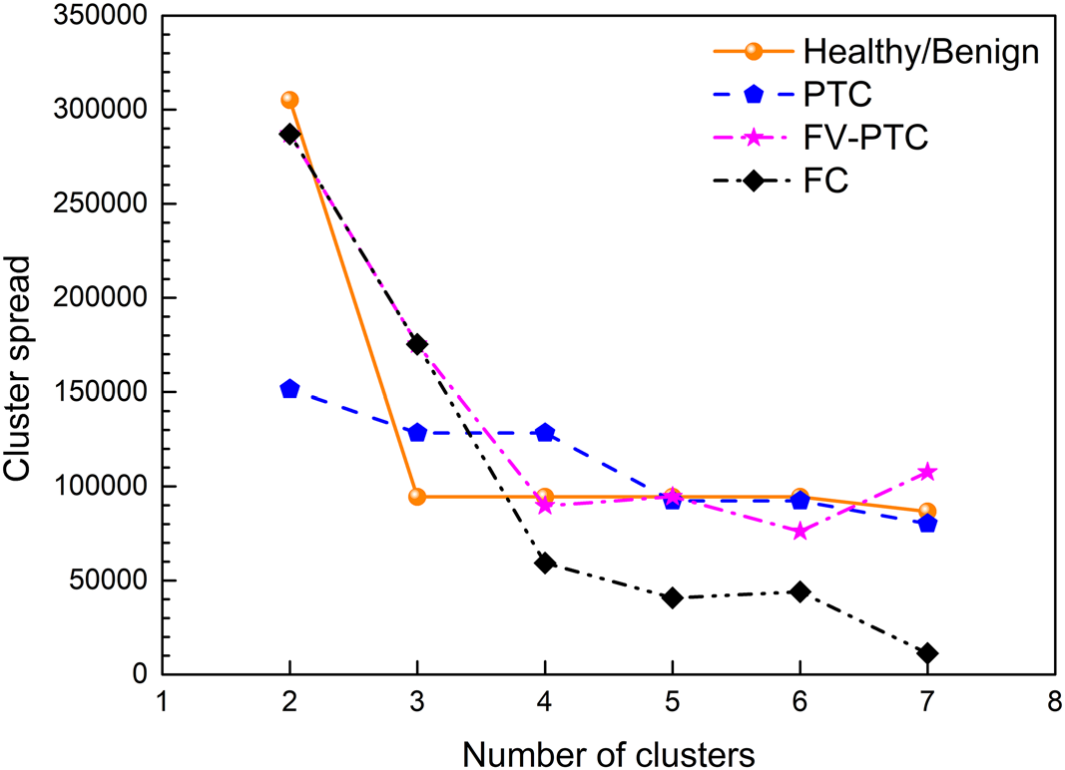
Spread of the individual K-means clusters as a function of their number. Data are reported by using the same colors as in Fig. 2 for the individual clusters. Notice that four clusters give the largest spread among the clusters.

The AHCA method aims at classifying observations (each Raman spectrum) into homogeneous groups (the clusters) based on the measured characteristics (Euclidean distance associated to each Raman spectrum). In particular, we have used the complete-linkage algorithm, in order to enhance differences among clusters. It works by pairing the most similar spectra (those showing the lowest dissimilarity value in the matrix) and then looking for the largest distance between such pair and the rest of the data. By iteratively repeating such an approach, a classification of spectra into well-separated groups is obtained and visualised as a dendrogram.

The KM method minimizes the variance within each cluster with respect to an a priori number of centroids, randomly distributed. This protocol starts by associating each data entry (Euclidean distance associated to each Raman spectra) to the cluster with the closest centroid. The centroids are iteratively updated towards convergence. The number of clusters (k) is an input of the routine. In order to choose the proper value of k, the most used strategy is the “elbow curve”, based on the explained variance within the entire dataset. This analysis in our case gives only two centroids, one for samples containing mainly carotenoids, and one for all other cases. Thus following the suggestion of the histology diagnosis, we have set k = 4. In order to verify the reliability of this choice, we have calculated the spread of each cluster with respect to its centroid as a function of k. Intestingly we find that k = 4 is the highest k-value with the lowest spread, as shown in Fig. 6.

We have tested the predictive accuracy of both AHCA and KM methods by generating a confusion matrix. This reports the comparison between the medical and spectroscopic diagnosis. The accuracy is defined as:

1 $$\begin{aligned} Accuracy = \frac{{True \, negative} +True \, positive}{number \, of \, total \, samples} \end{aligned}$$

### Biochemical analysis

Frozen thyroids have been weighted, homogenized and sonicated in lysis buffer (20 mM Tris-HCl pH 8.0, 137 mM NaCl, 10 mM EDTA, 10% glycerol (v/v), 1% Triton X-100 (v/v), and protease inhibitors). Twenty-five micrograms of protein extracts, previously quantified with the Bradford assay (Bio-Rad, Hercules, CA), have been resolved by 15% SDS-PAGE and transferred onto PVDF membranes (Bio-Rad). After blocking with 3% BSA (w/v) dissolved in TBS buffer/ 0.5% Tween-20 (v/v), membranes have been probed overnight at $4^\circ \hbox {C}$ with anti-cytochrome *c* (mouse mAb, sc-13560; Santa Cruz Biotechnology, Dallas, TX, USA) and anti-actin (mouse mAb, sc-47778; Santa Cruz Biotechnology) primary antibodies. Membranes have been then incubated for 1 h at room temperature with anti-mouse HRP-conjugated secondary antibody (Bio-Rad). All the experiments have been repeated trice. Blots have been acquired and processed using the ChemiDoc Imaging system (Bio-Rad). Cytochrome *c* quantification has been performed using the Image Lab software (version 6.0.1, Bio-Rad Laboratories). Results are shown as mean ± standard deviation (SD); the statistical significance of differences has been tested using the Student’s *t* test (GraphPad Prism, version 6.01).

### Ethics statement

The study was approved by the Ethical Committee of the University of Rome “Campus Biomedico” (UCBM) (prot. 33.15 TS ComEt CBM and prot. 31/19 PAR ComEt CBM). The informed consent was collected from patients before surgery. Enrolled patients were recorded in a codified file with an anonymous ID code, which was registered in the software database of the Pathology Unit of the UCBM. All experiments were performed in full accordance with the principle of Good Clinical Practice (GCP) and the ethical principles contained in the current version of the Declaration of Helsinki.

## Supplementary material

Supplementary Information.
