# Supplementary material for: Thyroid cancer diagnosis by Raman spectroscopy
Source: Sci Rep. 2020 Aug 7;10:13342. doi: 10.1038/s41598-020-70165-0 (PMC7414870; doi:10.1038/s41598-020-70165-0)
Supplement: Supplementary file 1 — Supplementary Information. [file 41598_2020_70165_MOESM1_ESM.pdf]

Thyroid Cancer Diagnosis by Raman Spectroscopy

Marco Sbroscia[1,2], Michael Di Gioacchino[1], Paolo Ascenzi[1], Pierfilippo Crucitti[3], Alessandra di Masi[1], Isabella Giovannoni[4], Filippo Longo[3], Davide Mariotti[1], Anda Mihaela Naciu[5], Andrea Palermo[5], Chiara Taffon[4], Martina Verri[4], Armida Sodo[1,*], Anna Crescenzi[4], and Maria Antonietta Ricci[1]

[1]Dipartimento di Scienze, Universitá degli Studi Roma Tre, via della Vasca Navale 84, 00146 Roma, Italy.
[2]present address: Dipartimento di Fisica, Sapienza Universitá di Roma, Piazzale A. Moro 5, 00185, Roma
[3]Unit of Thoracic Surgery, Campus Bio-Medico University, Roma, Italy
[4]Pathology Unit, Campus Biomedico University Hospital, Roma, Italy
[5]Unit of Endocrinology and Diabetes, Campus Bio-Medico University, Roma, Italy
[*]e-mail: armida.sodo@uniroma3.it

References

1. Tessler, F. N. *et al.* Acr thyroid imaging, reporting and data system (ti-rads): White paper of the acr ti-rads committee. *J. Am. Coll. Radiol.* **14**, 587 – 595, DOI: https://doi.org/10.1016/j.jacr.2017.01.046 (2017).

Supplementary information

Table 1. List of all the samples investigated by Raman Spectroscopy, along with clinical information. US Classification is done according to Tessler et al.[1]. Labels PTC, FV-PTC, FC, FA denote Papillary thyroid carcinoma, Follicular variant papillary thyroid carcinoma, Follicular thyroid carcinoma, Follicular thyroid adenoma, respetively. Label Oxy in the histology diagnosis refers to Oncocytic variants. Label HTT refers to Hyalinizing Trabecular Tumor. Finally, N.A. means not applicable. The superscript ** labels the samples used to generate the reference Raman spectra reported in Fig. **??**. Sample TIR59 is inadequate for Raman spectroscopy investigation: it was indeed too thin and the spectrum was dominated by the signal from the glass slide.

Samples	Gender	Age at diagnosis	US Classification (TI-RADS)[1]	Histology diagnosis	Raman Cluster	Immunochemistry diagnosis revision
TIR43	F	42	4	FA Oxy	FC	FA with altered markers
TIR44	M	62	3	FA	PTC	FA with altered markers
TIR45	F	77	4	PTC + FA Oxy	FC	PTC + FA Oxy
TIR46	F	42	5	PTC	PTC	
TIR47**	M	52	4	FC Oxy	FC	
TIR48	F	43	4	PTC Oxy	PTC	
TIR 49	F	45	4	PTC	PTC	
TIR50	F	30	5	PTC	PTC	
TIR51**	M	58	4	PTC	PTC	
TIR52	M	44	4	a) PTC<br>b) FC	a)PTC<br>b) FC	
TIR53	F	27	4	FA Oxy	Benign	
TIR55**	M	74	4	FC Oxy	FC	
TIR56**	F	43	4	FV-PTC	FV-PTC	
TIR57	F	47	4	FV-PTC	Benign	FV-PTC
TIR58**	F	38	4	PTC	PTC	
TIR59	F	32	4	FA Oxy	Inadequate	
TIR60	F	47	4	PTC	PTC	
TIR61	F	25	4	PTC Oxy	FC	PTC Oxy
TIR62	M	51	4	FA	Benign	
TIR63	F	31	3	FV-PTC	FV-PTC	
TIR64	F	65	3	FA	PTC	FA with micro PTC
TIR68	F	24	4	PTC	PTC	
TIR70	F	48	4	PTC	PTC	
TIR71	M	45	4	FA Oxy	FV-PTC	FA with altered markers
TIR72	F	32	4	PTC	PTC	
TIR73	F	58	4	HTT	PTC	HTT and PTC share common features
TIR74	M	39	3	FA Oxy	Benign	
TIR75	F	37	4	FV-PTC	PTC	FV-PTC
TIR76	F	42	5	PTC	FV-PTC	
TIR77	M	45	4	PTC	FV-PTC	
Hea 43	F	42	N.A.	Healthy	Healthy	
Hea46	F	42	N.A.	Healthy	Healthy	
Hea48	F	43	N.A.	Healthy	Healthy	
Hea49**	F	45	N.A.	Healthy	Healthy	
Hea50	F	30	N.A.	Healthy	Healthy	
Hea52	M	44	N.A.	Healthy	Healthy	
Hea56	F	43	N. A.	Healthy	FV-PTC	Near tumor area
Hea 57	F	47	N.A.	Healthy	Healthy	
Hea62	M	51	N.A.	Healthy	Healthy	
Hea63	F	31	N.A.	Healthy	FV-PTC	Near tumor area
Hea64**	F	65	N.A.	Healthy	Healthy	
Hea68	F	24	N.A.	Healthy	Healthy	
Hea70	F	48	N.A.	Healthy	Healthy	
Hea72	F	32	N. A.	Thyroiditis/Benign	FV-PTC	Near tumor area
Hea76	F	42	N.A.	Healthy	Healthy	

Supplementary Figure 1

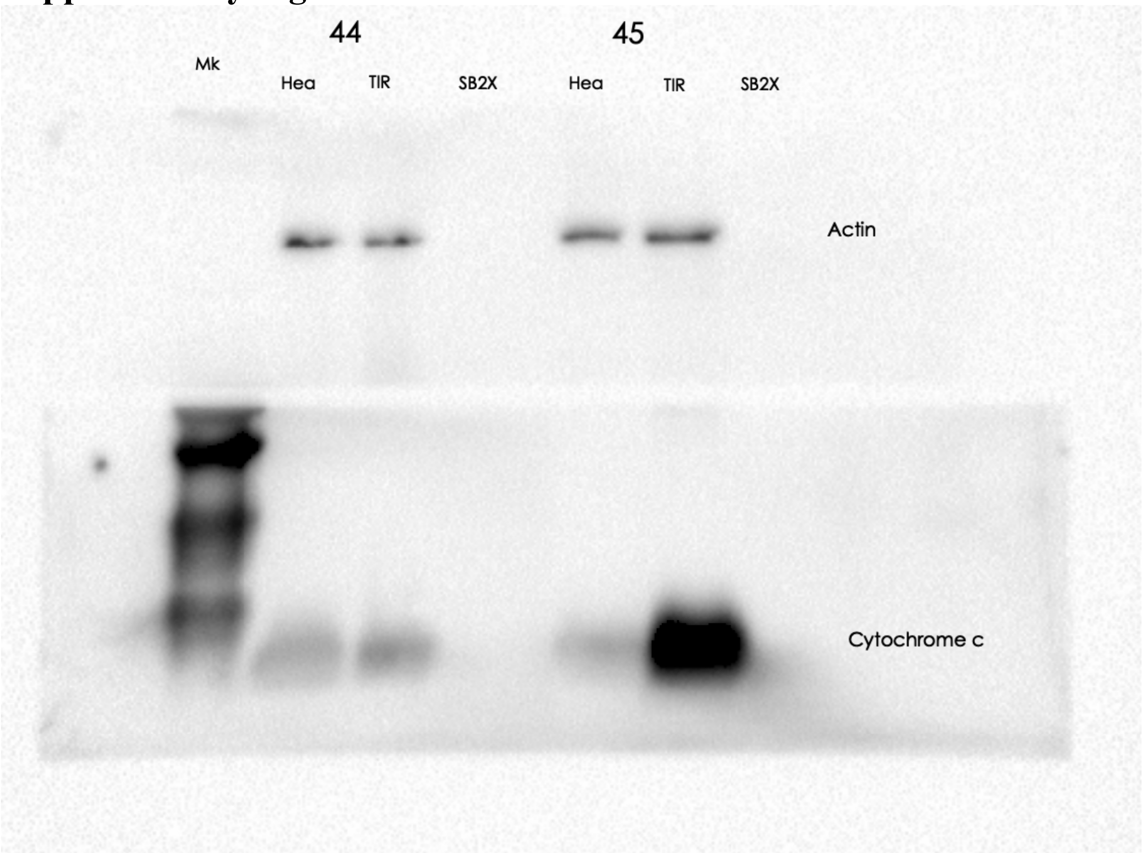

Supplementary Figure 1. Original images of data reported in Figure 5 with respect to patients 44 and 45. Filters were blotted with cytochrome *c* and actin primary antibodies. Images have been gathered at the same time. Mk, Marker; Hea, healthy tissue; TIR, pathological tissue; SB2X, sample buffer 2X.

Supplementary Figure 2

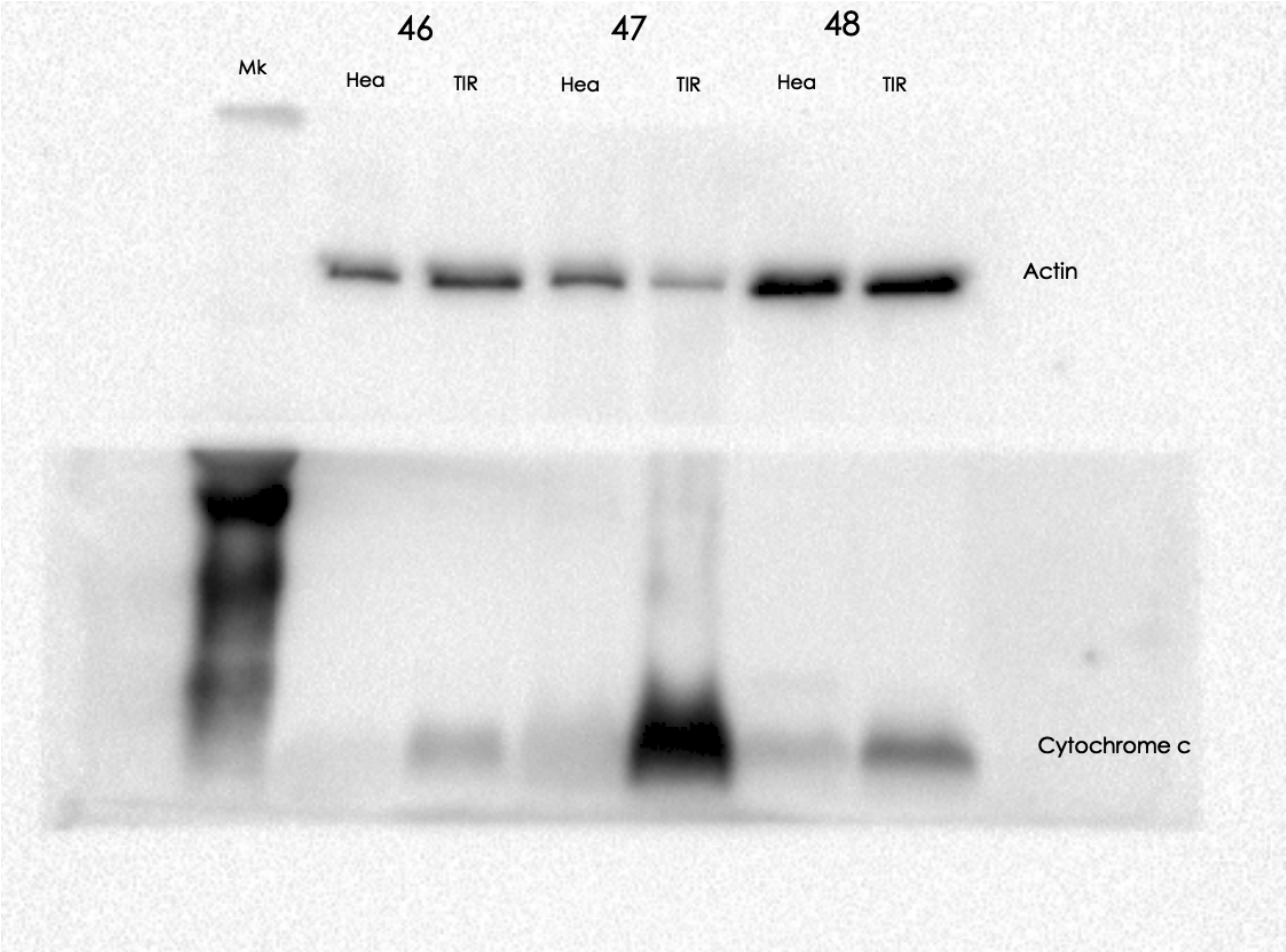

Supplementary Figure 2. Original images of data reported in Figure 5 with respect to patients 46, 47, and 48. Filters were blotted with cytochrome *c* and actin primary antibodies. Images have been gathered at the same time. Mk, Marker; Hea, healthy tissue; TIR, pathological tissue.
